# Study protocol on effectiveness of a holistic lifestyle intervention using eHealth system for older adults with metabolic syndrome and psychosomatic symptoms: a randomized controlled trial

**DOI:** 10.1186/s12877-025-06613-1

**Published:** 2025-12-29

**Authors:** Karen Siu-Lan Cheung, Bobo Hi-Po Lau, Angela Y. M. Leung

**Affiliations:** 1 School of Nursing, The Hong Kong Polytechnic University, Hong Kong SAR, China; 2 WHO Collaborating Centre for Community Health Services, School of Nursing, The Hong Kong Polytechnic University, Hong Kong SAR, China; 3 Mindlink Research Centre Limited, Hong Kong SAR, China; 4 Department of Counselling and Psychology, Hong Kong Shue Yan University, Hong Kong SAR, China; 5 Research Institute for Smart Ageing (RISA), The Hong Kong Polytechnic University, Hong Kong SAR, China

**Keywords:** Elders, MetS, Psychosomatic symptoms, Lifestyle intervention, EHealth system, Weight loss

## Abstract

**Background:**

Metabolic syndrome (MetS) is increasingly prevalent worldwide, characterized by hormonal imbalances and psychosomatic issues that often result in chronic pain and fatigue, and are associated with a heightened risk of depression and anxiety. While previous lifestyle interventions on MetS have shown health benefits, they often lack sustainability and neglect psychosomatic aspects. This highlights the necessity for innovative treatment protocols that can address these complexities. This study aims to examine the effects of the digital Holistic Healthy Life Education (e2HLE) intervention on various outcomes among community-dwelling older adults with MetS and psychosomatic symptoms in Hong Kong.

**Methods:**

A waitlist randomized controlled trial featuring two parallel arms—an experimental group (EG) and a waitlist control group (WLCG)—will recruit 150 participants aged 60 and older with MetS and psychosomatic symptoms from five elderly centers. Participants will be randomly assigned to either the EG or WLCG. The 12-week intervention comprises weekly theme-based sessions focused on self-care, whole food plant-based diets, physical activity, self-administered acupressure, and mindfulness practices. These components are supported by an eHealth system that offers educational materials and facilitates self-monitoring of bio-signals and lifestyle changes, along with a comprehensive booklet. A two-day workshop supported by a multidisciplinary team will be conducted prior to the intervention. The intervention strategies utilize a combination of modules, incorporating in-person individual and group meetings, tele-counseling, technology-driven support groups, and buddy-assisted health coaching. A mixed-methods approach will be employed, with EG participants undergoing pretest and posttest assessments through surveys, health examinations, and blood tests, supplemented by qualitative process evaluations. WLCG participants will receive usual care for the first three months before cross over to the intervention arm. Data will be collected between baseline and the 12-week follow-up, with quantitative analysis conducted using SPSS and generalized estimating equations models.

**Discussion:**

This study is the first to investigate weight loss and alleviate cardio-metabolic risk factors and psychosomatic impacts among older adults in a Chinese context, guided by the concept of intrinsic capacity. If the intervention proves more effective than the control group, it could facilitate broader implementation in community settings and enhance theory-building and clinical practice.

**Trial registration number and date:**

ClinicalTrials.gov ID: NCT06499961. First submitted 2024-05-7.

**Supplementary Information:**

The online version contains supplementary material available at 10.1186/s12877-025-06613-1.

## Introduction

Stroke and cardiovascular disease (CVD) risk factors remain significant global health challenges [1]. Metabolic syndrome (MetS), affecting about 20.1% of the global population, is a major concern due to its strong link to CVD [2, 3]. MetS is defined as a cluster of cardio-metabolic risk factors, including diabetes, abdominal obesity, hypertension, and hyperlipidemia [4]. It reflects hormonal imbalances and psychosomatic issues [5], frequently resulting in persistent somatic symptoms such as pain and fatigue [6]. These symptoms are associated with an increased risk of depression and anxiety [7], a concern that has become particularly pronounced in the aftermath of the COVID-19 pandemic [8]. The interplay between MetS and psychosomatic symptoms presents a significant threat to health and mortality risk, deserving particular attention. Hong Kong is no exception.

Older adults in Hong Kong are living longer but with worsening health [9]. The number of individuals aged 65 and over with chronic conditions—such as hypertension, diabetes, persistent pain and depression—has nearly tripled from 340,900 in 1999 to 1.01 million in 2020/21 [10, 11]. In response, a Primary Healthcare Blueprint was created to shift from a traditional medical model to a prevention-oriented, community-based approach aimed at improving overall health [12]. This blueprint recognizes the importance of behavioral management of CVD risk factors and lifestyle modifications, aligning with the World Health Organization’s (WHO) model of healthy aging and the Integrated Care for Older People (ICOPE) framework, which focuses on enhancing Intrinsic capacity (IC) in areas like cognition, functioning, physical health, psychological well-being, nutrition and sensory capabilities [13].

## Background

Many cases of MetS stem from preventable health-related behaviors and lifestyle practices [14]. Obesity is a major component of MetS, closely linked to its other risk factors [15], making weight loss a primary treatment and prevention goal [16]. Research shows that frequent self-monitoring of food intake correlates with greater weight loss [17]. Structured behavioral interventions, involving regular support and feedback, can achieve meaningful weight loss of 5% [18], regardless of specific dietary or activity components [19]. While body weight is widely regarded as a robust indicator of MetS, other metrics like cardiopulmonary fitness and cardio-metabolic parameters should also be considered [20]. Public health concerns emphasize the need for a holistic approach addressing psychosocial and lifestyle factors in preventing and managing CVD risk [21].

Psychological distress is a significant risk factor for both MetS and CVD [22]. A meta-analysis shows that high perceived stress increases the risk of coronary heart disease (CHD) [23]. Individuals with MetS often report lower quality of life and higher depressive symptoms [24]. Increased implicit negative affect and reduced heart rate variability (HRV), which indicate heart conditions and mental health issues, are associated with a higher risk of MetS [25]. MetS also involves autonomic nervous system dysfunction, leading to insulin secretion problems and glucose intolerance, as evidenced by delayed heart rate recovery (HRR) [26]. Over half of CHD patients experience persistent somatic pain in limbs, yet treatment options are limited [27]. Given the impact of psychosocial factors and somatic symptoms on MetS and CVD, there is an urgent need for clinical trials to assess mind-body therapies [28] and to develop personalized treatment protocols for managing MetS in conjunction with psychosomatic symptoms [5].

### Whole food plant-based diets

Combining pharmacotherapy with lifestyle changes leads to greater health benefits than medical treatments alone [29]. A whole food plant-based (WFPB) diet is increasingly recognized for its role in promoting health, reducing environmental impact, and improving animal welfare [30]. Evidence links high consumption of whole plant foods to a lower risk of conditions like CVD, stroke, metabolic dysfunction, and certain cancers [31–38]. These diets also enhance emotional and physical well-being [39]. A high-quality WFPB diet is crucial for weight reduction and improving high-density lipoprotein cholesterol (HDL-C) levels [40] and may help alleviate depressive symptoms [41]. Most research treats WFPB diets as a whole, overlooking how diet quality and specific food types affect their benefits against MetS [42]. While the Mediterranean and DASH diets have been extensively studied, there is a notable paucity of randomized controlled trials evaluating the effectiveness of WFPB diets for the treatment of MetS [43].

### Physical activity

Evidence suggests that regular, moderate-intensity physical activity (PA) is strongly associated with a lower risk of MetS [44]. PA improves HDL-C and triglyceride levels [45] and is linked to better quality of life, enhanced insulin sensitivity, and improved lipid profiles. It also aids in weight loss, protects bone mass, and improves body composition [46]. Beyond physical benefits, PA enhances mental health, boosting psychological well-being and sleep quality in older adults [47]. However, a meta-review indicates that PA’s effects on other CVD risk factors are less consistent, whether used alone or with dietary apps [48].

### Self-administered acupressure

Acupressure, a non-invasive treatment from Traditional Chinese Medicine (TCM), is effective in reducing stress [49] and MetS [50], as well as significantly lowering self-rated pain and anxiety [51]. Evidence supports the effectiveness of self-acupressure for pain relief and improved sleep quality in older adults [52] and those with CHD [53]. While self-administered acupressure is gaining popularity for managing MetS and CVD risk factors, further studies are needed to confirm its effects on glycosylated hemoglobin [54].

### Mindfulness

Numerous studies show that mindfulness-based interventions effectively address psychological conditions such as anxiety, stress, chronic pain, and depression, while also enhancing overall health [55, 56]. Mindfulness has been linked to eating disorders and MetS, with higher mindfulness associated with lower obesity rates [57]. Trait mindfulness correlates with a lower prevalence of MetS in both depressive and non-depressive individuals [58]. While mind-body practices improve MetS risk factors [59], adding mindfulness to diet-exercise programs has not shown significant weight loss benefits [60]. A mindfulness app combined with a comprehensive lifestyle intervention can help control eating but has not yet significantly impacted weight loss [61].

### Lifestyle modification programs

Interventions combining PA and mindfulness have proven particularly effective in improving health outcomes and quality of life, enhancing mental health and psychological well-being more than either approach alone [62]. The ongoing U.S. multi-site enhanced life modification trial targets healthy eating, PA, and mindfulness to slow the progression of MetS. While previous lifestyle interventions for MetS have shown health benefits, these often lack sustainability, highlighting the need for innovative treatment approaches [63].

### Ehealth interventions

Structured lifestyle modification programs significantly improve health outcomes for individuals with CVD risk factors. However, simply providing information about healthy habits can sometimes lead to adverse effects, such as increased Body mass index (BMI) and blood pressure [64]. In recent years, technology-based tools for monitoring dietary and physical activity patterns have proliferated [65]. Evidence shows that eHealth interventions with app support are more effective in reducing body weight, lowering the risk of MetS progression, and promoting healthier lifestyles [66, 67]. While these interventions can reduce weight and perceived stress, their impact on diet quality and PA needs further exploration [68]. Low compliance is a common challenge in obesity management [69], and employing buddy-assisted and health coaching techniques is essential for effective multi-component interventions [70]. These findings highlight the importance of considering intervention modalities, techniques, and participant characteristics in program implementation.

In Hong Kong, community-based RCTs have demonstrated that lifestyle modification programs can lead to clinically significant weight loss of 5–10% and help prevent diabetes in overweight and obese young and middle-aged adults [71]. However, females, particularly older adults, are more prone to abdominal pain as a psychosomatic symptom [72], exacerbated by factors such as depression, anxiety, and limited vaccine doses, especially post-COVID-19 [73]. While self-administered acupressure has shown effectiveness in alleviating anxiety and pain [74], research on its physiological effects remains inconsistent [75], highlighting the need for more rigorous studies [75]. Similarly, although higher plant protein intake is linked to improved physical performance in females [76], the majority of studies have primarily concentrated on middle-aged and older adults with normal functions [77]. There is a scarcity of clinical trials in Hong Kong that evaluate WFPB diets in conjunction with IC concepts [77]. Additionally, research is limited on how MetS risk factors affect psychosomatic health and the role of interdisciplinary collaboration in multimodal lifestyle interventions aimed at reducing cardio-metabolic risks and enhancing overall well-being.

## Methods

### Study design, objective and hypotheses

**Fig. 1:**
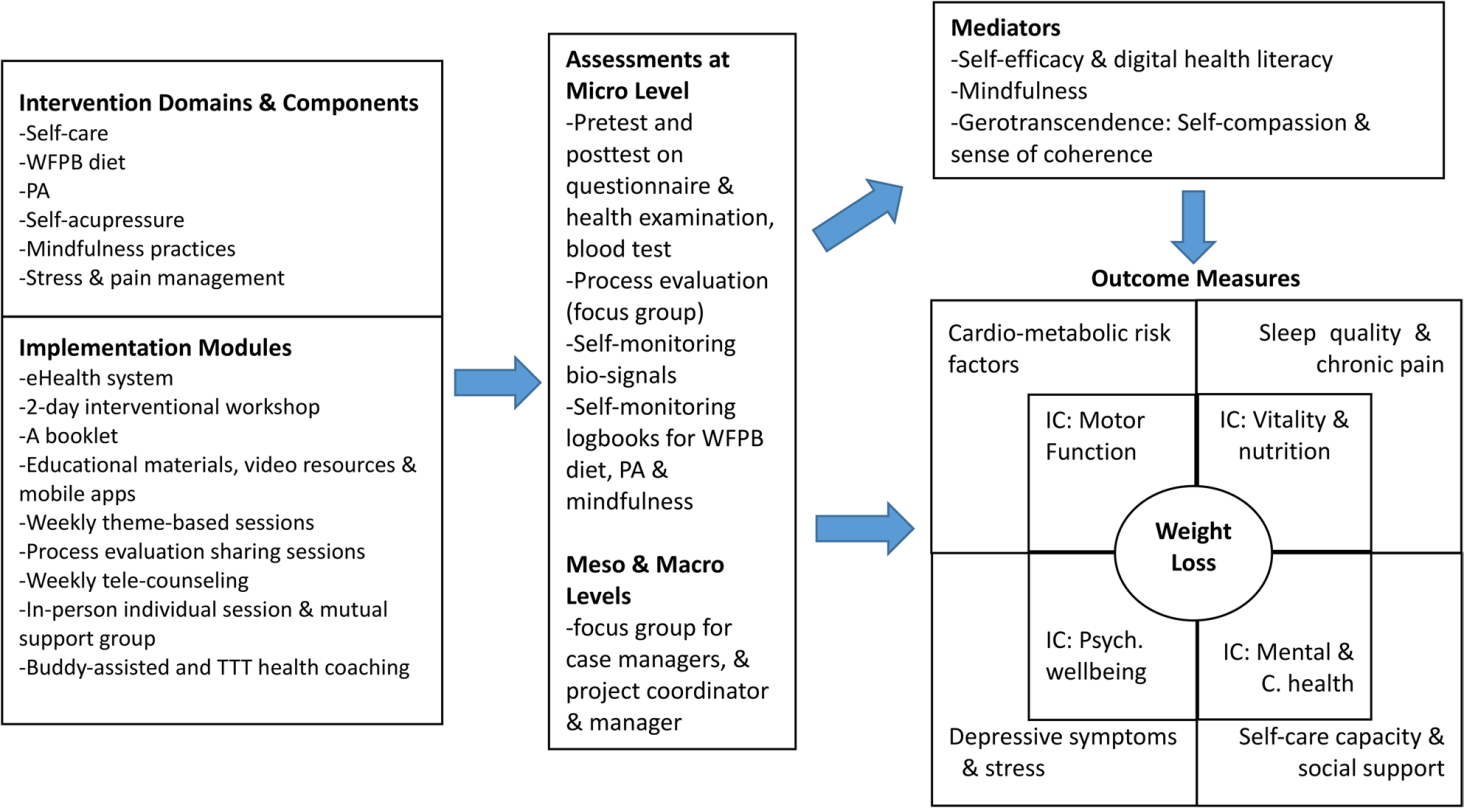
Hypothesized pathway and outcome measures framed with the multidimensional concept of intrinsic capacity (IC) alongside a multi-component evaluation framework for the e2HLE intervention

**Fig. 2:**
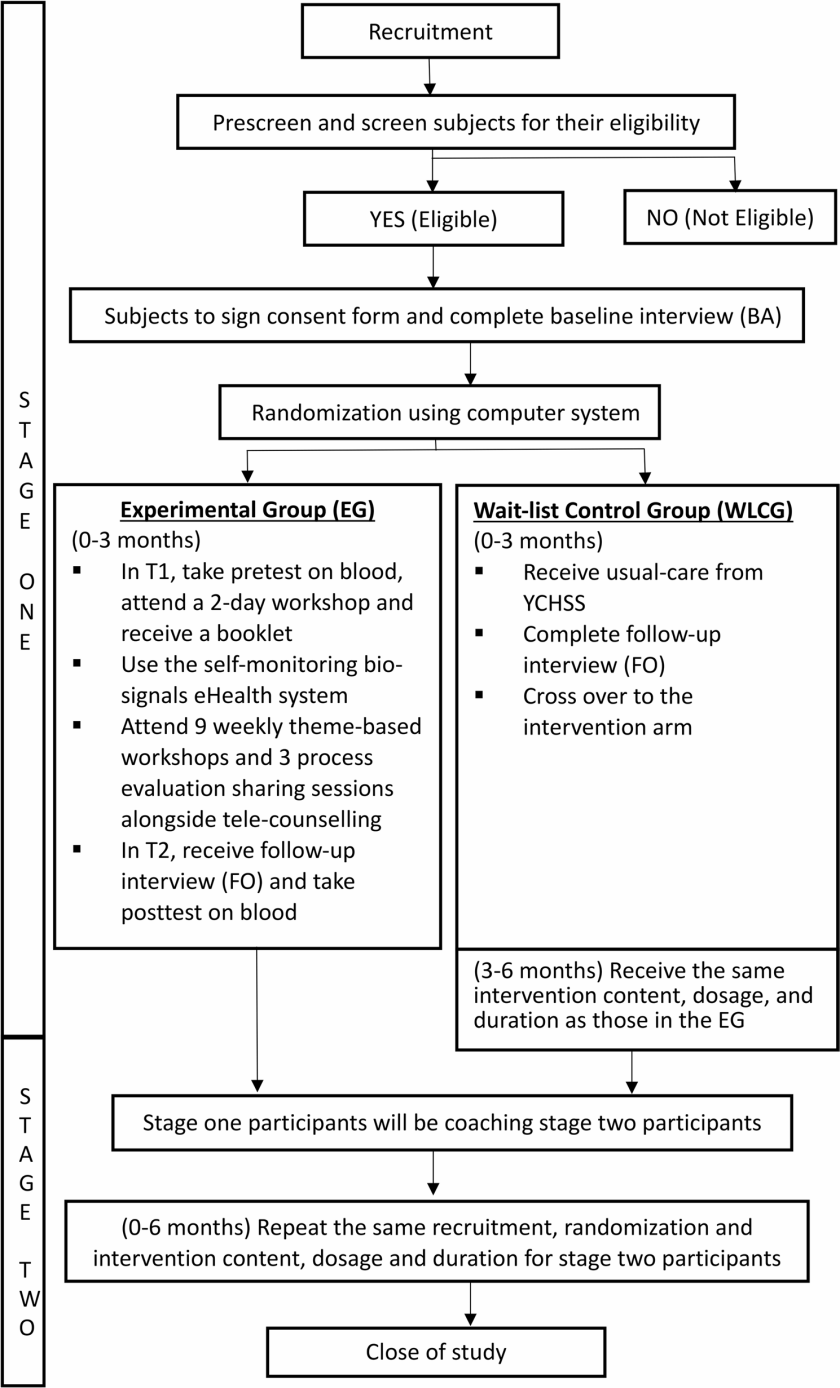
The flowchart of subject recruitment and intervention by dual

This study aims to examine the effects of the e2HLE intervention, which employs a home-based self-monitoring bio-signals eHealth system (referred to as the eHealth system), on various outcomes for community-dwelling older adults with MetS and psychosomatic symptoms in Hong Kong. Figure 1 illustrates the selection of outcome measures, guided by a hypothesized pathway that integrates the multidimensional concept of IC within a multi-component evaluation framework. The protocol of the present study received approval from the university and the selected elderly centers in Hong Kong. A parallel group, 1:1 randomized controlled trial design is employed, featuring two arms: an EG and a WLCG. Given the manpower and capacity constraints at the elderly centers, this study will be implemented in a dual-stage approach, as illustrated in Fig. 2.

We hypothesize that EG participants will experience significant outcomes, including weight loss (primary outcome). Considering secondary outcome measures, we expect improvements in IC, focusing on the four domains, namely motor function, vitality (nutrition), psychological well-being and mental and cardiac health and enhancements in at least two cardio-metabolic risk factors, which may include waist circumference, blood pressure, BMI, visceral fat, and blood test results (such as HbA1c, LDL cholesterol levels, and homocysteine). These measures also include improvements in sleep quality and chronic pain, self-care capacity and social support, and reductions in depressive symptoms and perceived stress levels. We also conjecture that the primary effect of weight loss and secondary outcomes will be mediated by several variables, including self-efficacy, digital health literacy, mindfulness, and gerotranscendence.

### Subjects and setting

Participants will be recruited from five elderly centers using posters and briefing sessions that offer various health promotion activities for local older adults. They will learn about the e2HLE intervention aimed at reducing MetS risk factors and alleviating psychosomatic symptoms. Interested individuals will be encouraged to register, and after completing the enrolment and eligibility screening, they will receive study consent forms and an authorization letter for the use of portrait. Pre-screening and screening will determine their eligibility for participation, with criteria outlined in Appendix A. To ensure consistent implementation of this multicomponent intervention across centers, we will standardize case worker training, provide detailed manuals and guidelines, and conduct regular monitoring. The eHealth system will facilitate centralized data collection and routine audits, which will be reviewed by the project coordinator and principal investigator. Feedback mechanisms, such as debriefings and surveys, will be employed to address site-specific challenges. Collectively, these strategies are designed to minimize heterogeneity and support reliable comparisons of outcomes.

### Sample size

Based on the literature review on previous similar RCTs [63], Cohen’s d ranges from 0.2 to 0.5 for small effect and from 0.5 to 0.8 and greater than 0.8, for medium and larger effects respectively. To detect a medium effect size of 0.5, 140 participants (126/1-0.1.1.1 = 140) are required (70 participants in each arm) to achieve a statistical power of 80% at the 5% significance level (alpha = 0.05, 2 tails). Based on the attrition rate of 8% in the pilot study at 3 months that was conducted in 2021/22, it is estimated to recruit 75 participants per group (i.e. 150 in total).

### Randomization, allocation, and concealment

Eligible participants at each elderly center will be randomly assigned to either an EG or a WLCG using a computer-generated allocation sequence with a 1:1 ratio, facilitated by Research Randomizer (Research Randomizer Version 4.0; www.randomizer.org). Participants living in the same household will be randomized as a single unit to reduce the influence of their dietary habits on each other. The allocation sequence will be documented and placed in sealed envelopes, which will be opened after participants complete a baseline questionnaire. They will then be informed of their group assignment and follow-up procedures. The healthcare team and research assistants will remain blind to group allocations. All participants in the EG will receive the intervention as per the established protocol, with follow-up sessions scheduled on different dates for each group to prevent contamination and maintain blinding for assessors.

### Intervention materials and theoretical framework

The eHealth system is designed to facilitate the implementation of a personalized, goal-based health care plan, enabling participants to monitor their health vitals at home on a weekly basis and engage in weekly tele-counseling sessions. The system comprises the following components: (1) temperature and oxygen monitoring (single-sided bio-sensing earbud for the right ear); (2) tablet; (3) FDA-certified Bluetooth blood pressure monitor; (4) docking station; (5) USB charging cable; (6) anti-static bag; (7) three ear gels (medium size); and (8) user manual. Additionally, a 5G data plan will be provided to all EG participants. The eHealth system captures seven key health vitals: heart rate, respiratory rate, core body temperature, oxygen saturation (SpO2), HRV as an indicator of mental health, HRR for cardiac health, and blood pressure.

**Fig. 3:**
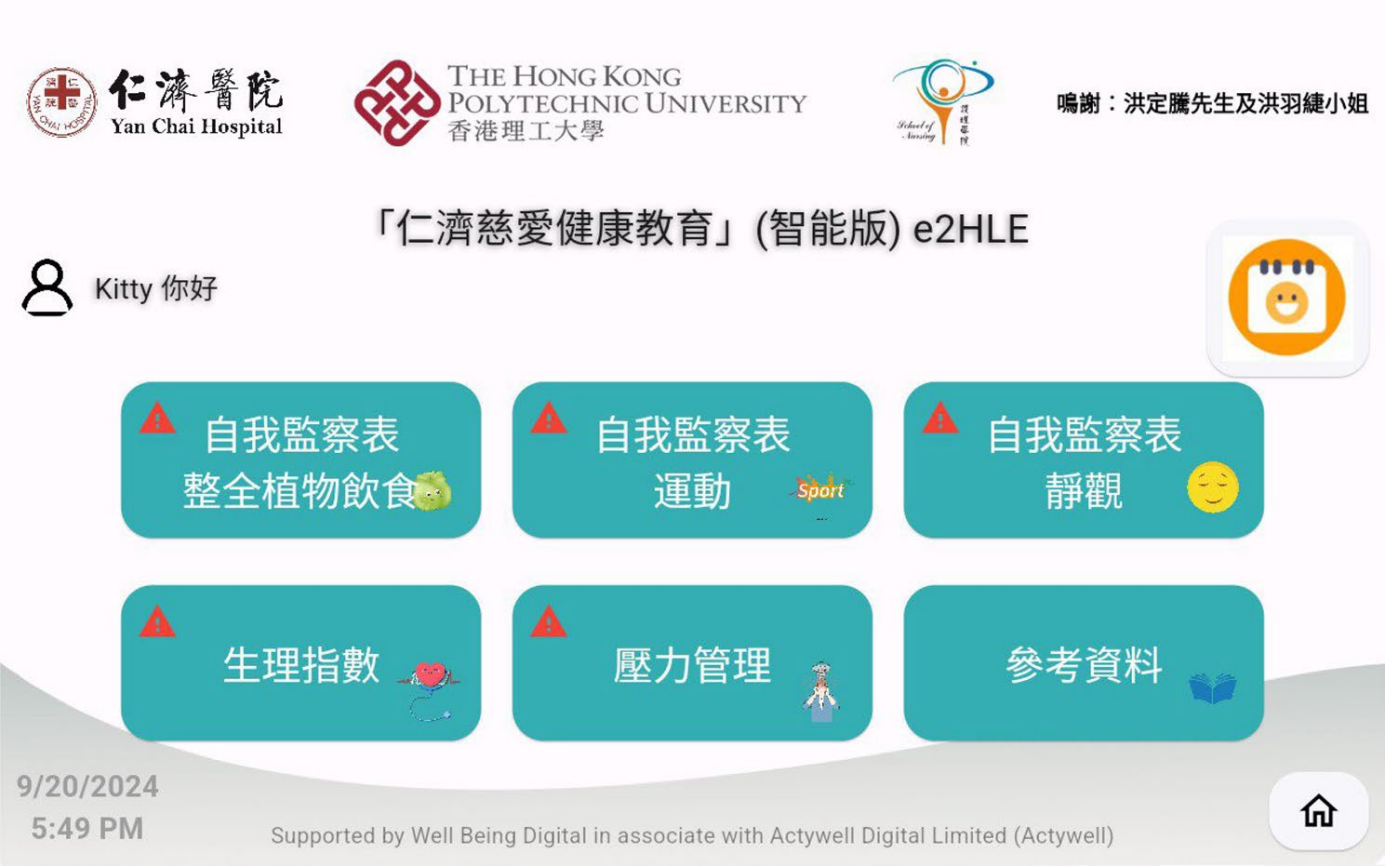
The launcher of the home-based self-monitoring bio-signals eHealth system for the e2HLE intervention

The front page of the eHealth system (Fig. 3) features a visually engaging user interface and user experience design that directs participants to six icons for self-monitoring logbooks and an educational section: (1) daily recording of WFPB dietary details; (2) documenting daily PA, accompanied by relevant video materials; (3) recording mindfulness practices; (4) monitoring and capturing seven health vitals; (5) documentation of a stress diary, thought records, and pleasant events; and (6) comprehensive information on MetS, psychosomatic symptoms and their health risk factors, as well as self-care strategies. Additionally, the eHealth system incorporates a calendar reminder feature for tele-counseling sessions and employs “cues to action,” highlighted in red with an exclamation mark, to promote self-initiated action and self-regulation among participants.

Four recognized heuristic frameworks commonly guide complex interventions: the RE-AIM framework, the Conceptual Model of Implementation Research, the Consolidated Framework for Implementation Research, and the PICO framework. Additionally, Parkinson et al. [78] proposed a multi-component evaluation framework that examines micro, meso, and macro levels to identify mechanisms for enhancing engagement and success in interventions. Our study utilizes a similar framework to assess the effectiveness, acceptability, fidelity, generalizability, sustainability, uptake, implementation costs, and scalability of a holistic lifestyle intervention in real-world social contexts (see Fig. 1).

### Adherence and fidelity

Throughout the 12-week intervention, self-monitoring logbooks within the eHealth system will be utilized to record WFPB diets, PA, and mindfulness practices (Appendix B). Dietary adherence will be quantified using a dietary adherence score derived from the food records. Initially, the food records will be analyzed using Food Processor software, version 11.1, developed by ESHA Research (Salem, Oregon, USA), to determine the nutrient and energy intake associated with each diet plan. Apart from the nutrient and energy intake, it is crucial to assess whether EG participants are consuming a diverse and abundant array of high quality whole plant-based foods, including leafy greens, cruciferous vegetables, rhizomes, solanaceous vegetables, legumes, beans, soy products, whole grains, nuts, and seeds.

Additionally, all EG participants are instructed to wear a single-sided earbud via the eHealth system while watching and following the exercise videos to facilitate simultaneous monitoring and capturing of their health vitals. Regarding mindfulness practices, participants will be required to log whether they engage in meditation, including the duration and types of meditation techniques practiced. Adherence to self-monitoring will be tracked over the course of the 12-week intervention. This adherence will be quantified by calculating the percentage of completed logbooks relative to the total number of logbooks targeted.

Starting in the third week of the intervention, EG participants are required to complete a stress management logbook within the eHealth system (Appendix B). Case managers (CMs), who are registered social workers, will facilitate discussions with EG participants to reinforce and remind them to apply the coping strategies they have learned.

The speech-to-text (STT) software is integrated into the tablet and facilitated by the eHealth system, allowing EG participants to input the aforementioned details either by audio or using a stylus pen for text entry. Audio recordings will be converted to text and transferred to the backend system, where CMs can retrieve and download these text files. CMs are required to review the logbooks and bio-signal files to implement and assess a goal-based health care plan in collaboration with EG participants prior to the weekly tele-counseling sessions. If participants experience any discomfort or difficulties during the intervention period, they will be referred to appropriate specialists.

Attendance for the 2-day workshop and the 12 weekly theme-based sessions will be monitored to ensure adherence to the intervention activities. Attendance will be calculated as a percentage of sessions attended relative to the total number of targeted sessions. Both attendance and self-monitoring will serve as common indicators of adherence. Previous research has indicated that when attendance distribution skews toward the higher end, attendance can be categorized into high (≥ 80%) or low (< 80%) adherence groups for analytical purposes [70].

### Weekly intervention evaluation notes by case managers

Throughout the 12-week intervention period, CMs are required to complete and submit a digital weekly intervention evaluation note to the project coordinator and principal investigator for regular review (Table 1). This process ensures that the structured intervention is delivered in accordance with the established protocol.

**Table 1:** The details of intervention evaluation notes by case managers

**Question**	**Details**
1.	Whether the weekly tele-counseling occurred outside the scheduled program
2.	The purpose of the counseling session
3.	Any health professionals or other individuals involved
4.	A record of which intervention components were discussed and reviewed
5.	Capture enablers and barriers to program implementation, troubleshooting issues related to the eHealth system
6.	Whether the recommendations were practiced, and the reasons for any failure to follow the advice
7.	The status of the participant's problems will be documented
8.	Whether these issues have worsened or improved since the beginning of the intervention, along with an assessment of the extent of any changes

### Intervention protocol

The intervention protocol is reported based on Template for Intervention Description and Replication (TIDieR) checklist and guide [79]. Prior to the commencement of the 12-week intervention, a two-day workshop will be conducted to introduce five key domains: (1) health risk appraisal and self-care; (2) PA and self-administrated acupressure; (3) emotion and stress management; (4) WFPB diet, nutrition, and disease relationships; and (5) meditation and mindfulness practices. This workshop is led by a multidisciplinary team comprising the principal investigator, registered TCM practitioner, senior physiotherapist (SPT), fitness trainer, registered dietitian, mindfulness instructor, senior registered nurse, and WFPB medical doctor. CMs are required to attend the workshop to familiarize themselves with the intervention content and the operations of the eHealth system. On the final half-day of the workshop, the eHealth system will be distributed to all EG participants. A briefing and hands-on training session will be organized by the information technology provider and technicians to demonstrate the operation of the eHealth system. All EG participants will be guided to learn self-monitoring logbooks to record their WFPB diet, physical exercise, mindfulness practices and a stress management logbook during the joint training and demonstration. An e2HLE booklet will be provided to all EG participants, encompassing a range of topics (Table 2).

**Table 2:** The details of the e2HLE booklet

**Component**	**Details**
PA and self-administrated acupressure	-The importance and key points of PA; warm-up exercise, stretching, balance training, muscle strength training, aerobic exercise, cool down exercise with photos, URL links and QR codes. -The origin, efficacy and operating techniques of acupressure and self-administrated acupressure methods for the twelve most common acupressure points (e.g., GV20 Baihui, GB20 Fengchi, GB21 Jianjing, BL52 Zhishi, LI10 Shousanli, ST36 Zusanli, LI4 Hegu, SP6 Sanyinjiao, BL40 Weizhong, KI1 Yongquan, etc.)
Meditation and mindfulness	-The theory, origin and scientific evidence of mindfulness; how meditation improves physical and mental health; meditation while walking, standing, sitting or lying down; understanding autopilot and its relationship to stress; be aware of distractions and stress and use your breath as an anchor; practice observing brain wave changes during breathing; how to practice mindfulness at home; apply what you have learned to re-energize your life; be kind to yourself; in times of stress, pause, breathe, and pour loving kindness into yourself; wise actions when stress rises; and meditation practice in life. -Mindfulness applications with QR codes (e.g., Pause and Breathe, Newlife 330, Mindful Flourishing, and Jockey Club "Peace and Awareness").
WFPB diets	-The concept of a whole food plant-based (WFPB) diet, common vegetarian traps, important sources of nutrients for vegetarians, holistic vegetarianism and disease, introduction to special vegetarian ingredients, WFPB recipes, and handling of ingredients. -Featuring WFPB plan and recipes for preparing meals, soups, snacks, and smoothies to be consumed throughout the week. The WFPB dietary plan is designed to provide appropriate levels of plant-based proteins, carbohydrates, and fats, along with essential nutritional components (e.g., vitamins and minerals) to support optimal health.

On the day of the intervention, CMs will provide approximately 30 min of counseling to all EG participants. They will be advised to gradually modify their dietary habits and establish a weekly routine that incorporates six designated exercise videos or other preferred physical activities, performed at a moderate-to-vigorous intensity for a minimum of 30 min per day, five days a week. Additionally, EG participants will receive guidance on self-administrated acupressure techniques, utilizing video clips that demonstrate the most common acupressure points, compiled by the TCM practitioner and SPT. Participants will also be instructed on how to use four meditation applications that are integrated into the eHealth system for meditation and mindfulness practices, allowing them to engage with these resources as needed.

EG: All EG participants will continue their prescribed medical treatments, if applicable. Throughout the 12-week intervention, participants will monitor their health vitals weekly and complete daily self-monitoring logbooks to record their WFPB diets, PA, and mindfulness practices. Table 3 presents the 12-week intervention sessions with three main components: (1) WFPB diet, (2) PA and self-administrated acupressure, and (3) meditation and mindfulness practices. They are required to attend nine weekly theme-based sessions. Each component consists of three sessions, with each session lasting 120 min. Additionally, there will be three process evaluation sharing sessions conducted in a focus group format, each lasting 60 min, resulting in a total of twelve sessions. During the weekly theme-based sessions, the WFPB diet component will cover various topics. Additional WFPB dietary consultation sessions with a registered dietitian can be arranged upon participant request throughout the entire intervention period. Regarding PA and self-administrated acupressure, EG participants will receive hands-on training in both physical exercise and fundamental self-acupressure techniques. Participants will also be encouraged to incorporate additional physical exercises into their routines as they progress. In the sessions related to meditation and mindfulness, various skills and techniques will be taught, as presented in Table 3.

**Table 3:** The 12-week intervention sessions with three main componentsThe 12-week intervention sessions with three main components

**12-session**	**Weekly theme-based sessions and process evaluation sharing sessions**
Sessions 1, Session 5, Session 9	**Component 1: WFPB diet ** Knowledge and evidence-based research of whole plant-based foods (i.e. whole grains, beans, legumes, fruits, vegetables, sea vegetables, seeds and nuts) along with rainbow colors, nutrition and disease relationships, reading food labels, healthy cooking methods, food preparation and storage, identifying common food traps, and avoiding animal-based and unhealthy foods (e.g. meats, milk, dairy products, eggs, refined carbohydrate, instant and convenient foods, processed and ultra-processed foods, intimation meats and vegetables, foods and sauces high in sodium, free sugar, trans-fats and saturated fats, leftovers, overcooked and deep-fried foods, double-stewed soup, shortening, margarine, palm and refined oils, Chinese sausage, salted fish and egg, fermented bean curd, sweet pastry and commercial beverages, alcohols, etc.). Calculate daily total energy intake and appropriate levels of macronutrients from non-animal sources, including carbohydrates, fats, and proteins, using the caloric application. Emphasize the consumption of whole plant-based foods, aligning with the basic tips to practice “Two Plus Three Every Day” – at least two servings of fruits and three servings of vegetables (https://www.chp.gov.hk/en/static/100011.html), as well as the importance of diet quality, dietary fiber, mono- and polyunsaturated fatty acids, polyphenols, phytochemicals, antioxidants, plant-based protein sources and fatty acids, and micronutrients (e.g., vitamin B12, vitamin D, potassium, iron, calcium etc.), along with maintaining adequate water and fluid intake. Problem-solving strategies for eating out and managing sickness during the intervention. Providing evidence-based WFBP video materials and sharing relevant meal preparation video clips. Cooking demonstrations will also be included and presented by a dietician.
Session 2, Session 6, Session 10	**Component 2: PA and self-administrated acupressure** Coach EG participants in performing exercises correctly. Coach to perform self-administrated acupressure correctly. Encourage to perform and retain any other preferred physical activities, such as brisk walking, swimming, yoga, badminton, Chinese dance, Tai Chi, or Qigong, etc. Instruct using exercise and acupressure videos, and emphasize the importance of wearing appropriate athletic attire and footwear to minimize the risk of injury.
Session 3, Session 7, Session 11	**Component 3: Meditation and mindfulness** Include inner climate examination, body scans, mindful stretching, and attentive eating practices. Include creating a meditation bottle, introducing a seven-step self-stabilization practice, identifying nine types of thought traps, and performing ten-finger gratitude exercises. Experience sound navigation, sensory exploration, and musical notes associated with hot springs.Demonstrate using four mindfulness applications.
Session 4, Session 8, Session 12	**Process evaluation sharing sessions** Discuss any barriers and facilitators they encountered related to intervention components; identify strategies for refining the intervention process; share their perspectives on enablers, and challenges encountered while using the eHealth system, including reasons for not achieving the expected weight loss.

EG participants’ family members, helpers or friends are invited to attend the theme-based sessions as observers or supporters at no cost, provided they give written informed consent. They may also assist participants who require support with digital literacy. Comprehensive step-by-step guides, hands-on training sessions, helpdesks, and dedicated IT staff will be available to assist with any digital challenges that may arise. This component is aligned with a “buddy-assisted health coaching approach” that enhances participant motivation and adherence to health-related behaviors, as encouragement from buddies can foster a sense of accountability and commitment to lifestyle changes. During the intervention, EG participants will have access to educational materials, videos, and meditation applications and maintain self-monitoring logbooks through the eHealth system.

During the process evaluation sharing sessions, EG participants will be organized into small groups of approximately 10 to 12 individuals (Table 3). Weekly individualized tele-counseling sessions by CMs, each lasting approximately 30 min, will be conducted. These sessions aim to increase motivation, review personalized care plans, and provide general support in making goal-based lifestyle changes and addressing challenges. Mutual support networking will be facilitated regularly between CMs and participants through WhatsApp or telephone communication. Each EG participant will receive a supermarket gift card valued at HKD200 (approximately USD25) as reimbursement for their time and registration fees, as well as an incentive for completing the 12-week intervention and participating in data collection.

WLCG: Participants will receive usual care from the elderly centers for the first three months. For ethical reasons, those in WLCG will crossover to the intervention arm after 12 weeks, receiving the same intervention content, dosage, and duration as the EG participants.

After completing stage one, all participants will attend a swearing-in ceremony where selected individuals will become “Smart Healthy Aging” ambassadors, coaching participants in stage two. This approach follows the Train-the-Trainer (TTT) model [80] and incorporates health coaching [69]. Key trained participants will share their insights, provide ongoing support to new participants, and facilitate the intervention process to build confidence and competence. They will receive a certificate of appreciation during the ceremony. The entire implementation process will be videotaped to create non-commercial documentary clips that aim to synthesize and disseminate evidence on the effectiveness of community-based behavior change interventions to a wider audience.

The evaluation of e2HLE is grounded in a whole-systems approach and examines three levels of the system: (i) the individual participant at the micro system level, focusing on effectiveness, process, implementation, and outcomes; (ii) the site or region at the meso system level, which considers the program context; and (iii) the overall program at the macro system level, assessing the potential for program sustainability at the organizational and/funder levels. In addition to focusing on the micro level, which examines individual participants, qualitative assessments will be conducted with CMs, experts as well as the project coordinator and social services department manager, to evaluate the meso and macro levels after the completion of both stages.

### Procedure of data collection

Eligible participants will be invited to join the study after providing signed consent following a detailed explanation of the study’s purpose and procedures. Baseline data (T1) will be collected by trained assessors. After the 12-week intervention, assessors who are blinded to group allocations will conduct physical assessments. Participants will complete questionnaire surveys, including health examinations, using a secure institutional account in Qualtrics to ensure data privacy. They will fill out the baseline questionnaire and health examination before randomization and participate in a follow-up interview approximately three months later (T2). A structured questionnaire will collect data, with only demographic information gathered at T1, and all measurements will be administered in Cantonese, consistent with previous Hong Kong studies. Appendix C details the outcome measures, assessment methods, and psychometric information.

### The primary outcome

Body weight will be measured using the same electronic weight scale in the community center.

### Secondary outcomes

The score range, direction and psychometric details of the secondary outcomes are presented in Appendix C. The secondary outcomes include enhancements in IC, specifically targeting four domains: motor function, vitality (nutrition), psychological well-being, and mental and cardiac health facilitated by the eHealth system. Additionally, improvements are anticipated in at least two of the cardio-metabolic risk factor profiles, including waist circumference, blood pressure, BMI, visceral fat, HbA1c, LDL cholesterol levels, and homocysteine (assessed through blood tests). Other expected outcomes encompass improved sleep quality and reduced chronic pain, decreased depressive symptoms and stress levels, and enhanced self-care capacity and social support.

### Pre- and post- blood tests

A total of 20 ml of blood will be drawn and tested by medical professionals in a designated laboratory. The blood tests will cover five main categories: (1) Albumin (nutritional marker); (2) CRP (inflammation marker); (3) Total blood cell count, creatinine, BUN, liver function tests, blood sugar levels, and HbA1c (indicators of anemia, diabetes, and organ function); (4) Total cholesterol, HDL, and LDL levels (cardiovascular disease risk factors); and (5) Homocysteine (indicating potential vitamin B-12 or folate deficiency). Individualized sessions lasting 15 to 20 min will be held for all EG participants after receiving their pretest and posttest blood reports, approximately 2 to 3 weeks after the intervention starts and ends. During these sessions, a registered dietitian and/or a WFPB medical doctor will provide explanations and advice for follow-up care. If any blood test results are abnormal, appropriate referrals for further treatment will be recommended.

### Mediators

This study hypothesizes that weight loss and its secondary outcomes are mediated by several factors, including self-efficacy, digital health literacy, mindfulness, and gerotranscendence. These elements collectively enhance an individual’s ability to adopt and maintain healthy behaviors. Higher self-efficacy helps individuals overcome obstacles and stay committed to weight loss goals. Digital health literacy is crucial in today’s tech-driven healthcare, enabling effective access to online health information and resources for informed decision-making. Mindfulness promotes awareness of thoughts and feelings related to food and body image, fostering healthier eating and emotional regulation. Gerotranscendence reflects a shift in perspective in older age, motivating individuals to prioritize their health. Together, these variables create a supportive framework that increases the likelihood of successful weight loss by addressing both psychological and practical aspects of behavior change (Fig. 1).

### Validity and reliability

All the selected measures demonstrated good reliability with Cronbach’s alpha >0.7 in past samples with local older adults and have been adopted for the local or Chinese context. For example, the Chinese version of the Pittsburgh Sleep Quality Index (CPSQI) is a reliable and valid instrument to assess subjective sleep quality. The CPSQI showed acceptable test-retest reliability with a coefficient of 0.85 for all subjects and 0.77 for primary insomniacs [81]. The General Self-Efficacy scale is commonly used to measure behaviors related to motivation, self-esteem, mental health, general well-being, and quality of life. The scale has demonstrated good internal consistency (α = 0.91) and criterion validity, making it suitable for investigating self-efficacy related issues in the Chinese population [82]. Any difference in baseline characteristics between groups will be accounted for in the statistical analysis.

### Data management

A project coordinator will help enroll participants and oversee the progress of the study, ensuring that participants’ rights and well-being are maintained. This individual will also ensure adherence to the study protocol, ethical standards, and regulatory requirements. Additionally, the coordinator will be responsible for reporting the proper operation of the eHealth system and the accurate recording of data and will monitor coding, security, and encrypted storage of all collected data.

### Planned data analysis

Data will be entered and analyzed using SPSS version 30. Demographic data will be summarized, with frequency and percentage for categorical variables, and mean and SD for continuous variables. Group difference at baseline will be compared using chi-squared tests for categorical variables and ANOVA for continuous variables. The Pearson correlation will be used to explore the relationship between weight loss and adherence scores. Changes in variables between baseline and the three-month follow-up will be compared using paired t-tests or Wilcoxon signed-rank tests as appropriate. To compare the mean changes in the continuous outcome variables including body weight, waist circumference, BMI, HRV and HRR, sleep quality, nutritional status, self-care capacity, perceived depressive symptoms and stress level, chronic pain among the two groups with the intention-to-treat principle. This approach ensures that all participants are included in the analysis as originally assigned, regardless of their level of compliance, thereby mitigating the impact of missing data. For missing values, methods like multiple imputation and sensitivity analyses will test the robustness of results. Adherence rates will also be analyzed alongside outcomes, using generalized estimating equations to account for repeated measures and reliably estimate intervention effects. Medication use for metabolic diseases and psychosomatic symptoms will be documented at baseline and follow-up, serving as a covariate in analyses to distinguish intervention effects. Subgroup analyses may compare outcomes between medicated and non-medicated participants. As age may significantly influence participants’ responsiveness to the intervention, conducting subgroup analyses by age cohort offers more specific insights into how the intervention may benefit older adults at different stages of aging. Post hoc pairwise comparisons of the changes in the outcome variables across the two study groups will be performed to compare the effectiveness of the EG and the WLCG. All the tests are two-sided and a *p* < 0.05 will be considered statistically significant.

Process evaluation and focus group data will be analyzed using qualitative content analysis. The process evaluation for individual participants and focus groups for intervention providers will be audio-recorded, with relevant portions transcribed verbatim into Chinese and subsequently cross-checked by research assistants and study investigators. Cantonese transcription will be facilitated by AI translation system. The qualitative analysis will employ an inductive thematic analysis approach [83].

## Discussion

To our knowledge, this study is the first to compare the effects of a holistic multi-component lifestyle intervention using a home-based self-monitoring eHealth system against usual care for community-dwelling older adults with MetS and psychosomatic symptoms in a Chinese context. The intervention includes self-care, WFPB diets, PA, self-administered acupressure, mindfulness practices, and stress and pain management. A balanced WFPB diet facilitates weight loss, improves lipid profiles, reduces inflammation, and supports overall health, which is crucial for managing MetS and psychosomatic symptoms.

Additionally, PA enhances insulin sensitivity, improves blood flow, and reduces body fat, while mindfulness reduces stress and psychological distress, supporting metabolic functions. Mindfulness practices align with Buddhist principles like non-attachment and compassion, promoting a sense of wholeness. Self-administered acupressure also alleviates stress and promotes relaxation, benefiting metabolic health and well-being. The effectiveness of interventions can vary by individual, but a multifaceted approach combining WFPB diet, PA, mindfulness, and acupressure may yield the best outcomes for managing MetS and psychosomatic symptoms.

The intervention is delivered through eHealth technology and includes a two-day workshop, a comprehensive booklet, educational resources, video materials, mobile apps, weekly themed sessions, process evaluation sharing, tele-counseling, and support groups. It combines in-person and group meetings with technology-driven methods, along with buddy-assisted and TTT health coaching. This approach promotes open communication to enhance older adults’ knowledge of nutrition and WFPB diets, while improving their diet, PA, acupressure, mindfulness self-efficacy, and coping skills for stress and pain. The intervention aligns with the WHO ICOPE framework, focusing on healthy aging, weight loss, and improving IC. A multi-component evaluation framework, using both quantitative and qualitative methods, supports comprehensive assessments of program effectiveness, quality, community impact, and outcomes.

A key strength of this study is the implementation of an eHealth system that allows participants to monitor their bio-signals at home, promoting self-care and self-management in line with goal-based self-regulation theory. This system enhances adherence through self-monitoring logbooks and features simplified functionality via STT software, making it accessible even for illiterate participants. Many older adults expressed a need for such technology during the pilot study in 2021/22, as concerns about usability and device compatibility often hinder their engagement with mobile phones. The digitization of the multi-component lifestyle intervention improves accessibility and flexibility, potentially increasing motivation for behavioral change.

The study also introduces a WFPB diet, trialed for the first time in a Chinese context, encouraging participants to consume a diverse range of high-quality plant foods. Given that changes in dietary habits require comprehensive, systematic guidance with well-defined objectives, participants will be supported by a registered dietitian in establishing realistic, personalised dietary goals, with adherence monitored through the submission of food diaries within the eHealth system. Furthermore, all EG participants will receive individual counselling following both pretest and posttest blood reports, enabling ongoing assessment of their progress, identification of challenges, and reinforcement of their commitment to healthy eating practices. This research aims to clarify the effects of a WFPB diet on metabolic health, emotional stability, psychological well-being, and pain relief, enhancing understanding of the relationship between MetS and psychosomatic effects.

Additionally, the intervention includes a buddy-assisted approach and TTT initiative, fostering social support and health coaching. This collaborative model promotes a communal health culture, normalizing positive behaviors and enhancing program sustainability as trained participants can effectively deliver the intervention to others.

Finally, the multidisciplinary team provides comprehensive support, tailoring interventions to individual needs. The program’s findings will offer valuable insights for service providers, healthcare teams, and policymakers, advancing the use of eHealth technology to promote healthy aging. By exploring the connections between IC, MetS, and psychosomatic symptoms, the study aims to deepen understanding of these mechanisms for theoretical and practical applications in clinical practice.

### Limitations

Given resource limitations and current trends in the literature, this study will implement a 12-week multi-component lifestyle intervention. While there is no gold standard for determining the optimal timeframe, this duration has been selected to balance effective outcome measurement with participant retention and aligns with the most commonly adopted timeframes in existing research. However, the maintenance effect will not be assessed within the scope of this study. Conducting the intervention in a natural setting presents several challenges. Participant recruitment is difficult, particularly for engaging working older adults with MetS and psychosomatic symptoms, as most potential participants are older individuals and retirees. Recruiting via center-based invitations often draws participants with favorable views of the institution, strong altruistic motivation, and flexible schedules. Previous internet-based surveys have reported over-representation of women, married, and well-educated individuals [84]. Consequently, there is a risk of participation bias associated with voluntary enrollment, which may particularly contribute to a gender imbalance by disproportionately increasing the number of female participants. To address this, we will track enrollment demographics and, where possible, conduct targeted outreach to boost male participation for better balance. Additionally, a high attrition rate is a concern, especially during the 12-week follow-up, as increased travel post-COVID-19 might affect adherence to behavior changes.

The home-based self-monitoring eHealth system has limitations, as it requires a docking station, which may hinder usability for active participants. To enhance accessibility, participants are encouraged to download relevant video materials and applications on their mobile devices. Regular software updates and bug fixing are anticipated, which may impact its performance and participants’ willingness to engage with the system.

Social support can be limited, as some participants may hesitate to invite friends or family members to sessions due to privacy concerns or fear of judgment. This underscores the need for strategies that promote open communication and reduce barriers to participation. While the TTT initiative is in place, its effectiveness is still being evaluated, and qualitative assessments will help gather feedback during the intervention’s stage two. However, further elaboration is required to enhance the internal validity of this approach.

A key component of our study focuses on WFPB diets, whereas fecal samples have yet to be collected for analysis. The relationship between WFPB diets and the gut microbiome, along with their underlying metabolic and inflammatory effects, remains largely underexplored [85]. Future research should explore how WFPB diets influence gut health and immune function in older adults.

Additionally, our study relies on self-reported stress and chronic pain, which are subjective measures. The absence of cortisol level assessments is a limitation, as cortisol is a key biomarker for stress and metabolic health. The eHealth system could assess stress through HRV, but future studies should incorporate cortisol assessments and other objective methodologies, such as neuroimaging and electromyography, to better evaluate psychosomatic symptoms and stress.

Lastly, this study addresses the complexities of changing eating habits and dietary patterns, emphasizing that individual dietary choices are heavily influenced by environmental and societal factors. The acceptability of a WFPB diet may be limited by various elements, including age, socioeconomic status, religion, living arrangements, family obligations, peer influences, health issues, food culture, self-control, and entrenched beliefs. To improve adherence and outcomes, WFPB dietary guidelines should be culturally adapted, and strategies should focus on incremental changes rather than drastic shifts in eating habits. As the study exclusively involved Chinese participants, further research is needed to determine the effectiveness of the intervention across other ethnic groups. Attention should be given to the sustainability of lifestyle interventions, including a maintenance phase to assess the long-term preservation of results.

## Conclusion

Sustained lifestyle changes require ongoing adjustments in mindsets and behaviors, emphasizing the need for a humanized approach and interdisciplinary collaboration to understand behavior and habit changes effectively. This study has important clinical and policy implications, including potential savings in manpower and resources. It promotes interoperability in data sharing and emphasizes a prevention-oriented self-care approach that transcends traditional health sector responses, ultimately enhancing care coordination. The e2HLE intervention, utilizing the eHealth system, focuses on advancements in prevention, innovation, technology, clinical research, and the assembly of multidisciplinary teams. If the e2HLE intervention is effective for the experimental group and positively impacts the wider community, it may be beneficial to scale up and implement the intervention in broader community settings or across different regions and countries.

## Supplementary Information

Supplementary Material 1

## Data Availability

No datasets were generated or analysed during the current study.

## Abbreviations

MetS: Metabolic syndrome
e2HLE: The digital Holistic Healthy Life Education
EG: Experimental group
WLCG: Waitlist control group
CVD: Cardiovascular disease
WHO: World Health Organization
ICOPE: Integrated Care for Older People
IC: Intrinsic capacity
CHD: Coronary heart disease
HRV: Heart rate variability
HRR: Heart rate recovery
WFPB: Whole food plant-based
HDL-C: High-density lipoprotein-cholesterol
PA: Physical activity
TCM: Traditional Chinese Medicine
BMI: Body mass index
SpO2: Oxygen saturation
CMs: Case managers
STT: Speech-to-text
SPT: Senior physiotherapist
TTT: Train-the-Trainer
CPSQI: Chinese version of the Pittsburgh Sleep Quality Index
BMR: Basal metabolic rate
CMNA: Chinese version of the Mini-Nutritional Assessment
CBPI: Chinese version of the Brief Pain Inventory
CGDS: Chinese version of the Geriatric Depression Scale
CPSS: Chinese version of the Perceived Stress Scale
CSCS: Chinese version of Self-Care Scale
CGSE: Chinese version of the Generalized Self-Efficacy Scale
CeHEAL: Chinese version of the eHealth Literacy Scale
CFFMQ: Chinese version of the Five Facet Mindfulness Questionnaire
CNonattachment: Chinese version of the Nonattachment Scale
CSCS-SF: Chinese version of the Self-Compassion Scale Short-Form
CSOC: Chinese version of the Sense of Coherence Scale

## Declarations

### Availability of data and materials

The datasets used and/or analyzed during the current study are available from the corresponding author on reasonable request.
