# Supplementary material for: Study protocol on effectiveness of a holistic lifestyle intervention using eHealth system for older adults with metabolic syndrome and psychosomatic symptoms: a randomized controlled trial
Source: BMC Geriatr. 2025 Dec 29;26:140. doi: 10.1186/s12877-025-06613-1 (PMC12859904; doi:10.1186/s12877-025-06613-1)
Supplement: Supplementary file 1 — Supplementary Material 1 [file 12877_2025_6613_MOESM1_ESM.docx]

# **Appendix A** Prescreening, Screening and Inclusion and Exclusion Criteria

Initial prescreening will be conducted using a simple health assessment, which includes criteria such as the presence of psychosomatic symptoms (pain or mood disturbances within the past six months), a BMI of ≥ 23.0 kg/m², and indicators of central obesity, specifically the presence of high blood pressure, hyperglycemia, or hypercholesterolemia. Participants will also undergo risk appraisal, mood and stress level assessments, and evaluations of self-care capabilities (feeding, bathing, dressing, and toileting). If participants exhibit difficulty understanding the interviewer's questions, the Chinese version of the Short Portable Mental Status Questionnaire (SPMSQ) [1] will be administered during the screening process.

*Inclusion criteria*

The inclusion criteria are those:

1. Aged 60 years old or above, able to use internet and tablet;
2. Education level of primary or above and understand Chinese and speak Cantonese;
3. Have central obesity (≥ 80cm for females; ≥ 90cm for males)and two of the following: e.g., hypertension (systolic blood pressure ≥130 or diastolic blood pressure ≥ 85 mmHg, or treatment of previously diagnosed hypertension), hyperglycaemia (fasting plasma glucose ≥ 100 mg/dL (5.6 mmol/L) or previously diagnosed type 2 diabetes), hyperlipidaemia (triglyceride concentrations ≥ 150 mg/dL (1.7 mmol/L), or treatment for this lipid abnormality); HDL cholesterol < 40 mg/dL (1.03 mmol/L) in males and < 50 mg/dL (1.29 mmol/L) in females, or treatment for this lipid abnormality),
4. BMI over 23;
5. and chronic pain or bad mood caused by the psychosomatic disorder for no less than six months and have at least 2 of the following distressing symptoms due to psychosomatic or pain and stress symptoms, including physical and mental agitation, feeling tired, often crying, feeling angry/depressed, feeling isolated from family and friends, feeling great stress, insomnia, health is worse than before.

*Exclusion criteria*

However, if subjects have the following criteria, they are not eligible and excluded, such as non-Chinese nationality; participating in any cancer treatment; will leave Hong Kong within a short period or the next three months; participating in another experiment; living in or planning to live in a facility-care home; cognitive difficulties (SPMSQ score equal to or lower than 4); and taking medications such as estrogenic, synthetic glucocorticoids, anti-steroids, and antiepileptic drugs which may affect cortisol levels.

**Appendix B** Self-monitoring Logbooks within the eHealth System

| **Component** | **Self-monitoring logbooks** |
| --- | --- |
| **WFPB diet** | -Input detailed information regarding their meals, including five designated meal categories: breakfast, lunch, dinner, snacks, and refreshments.  -Select an appropriate time frame for each meal from three available time slots (i.e., 00:00–11:59, 12:00–16:59, and 17:00–23:59).  -Indicate whether the meals were prepared by themselves; if not, they must provide the name and type of the restaurant, along with details of the meals, such as the types and quantities of food and beverages consumed. |
| **Physical activity** | -Record their daily physical exercises information such as date, time, duration and types of exercises performed.  -Watch and follow the exercise videos to facilitate simultaneous monitoring and capturing of their health vitals. |
| **Mindfulness** | -Log whether they engaged in meditation via the eHealth system  -Record detailed information, including date, time, duration and types of meditation techniques practiced, such as body scan, mindful smiling, signal breath, meditation with stretching, and five-sense experiences. |
| **A stress management and behavioral activation**  **logbook** (Starting in the third week of the intervention) | **Stress diary:** utilize a stress thermometer scale, which ranges from 0 (no stress) to 5 (very stressful), to rate their stress before and after employing stress-reducing techniques. They will be instructed to describe specific situations that triggered their stress, including details about the context, their emotional responses, and the effectiveness of the stress-reducing methods they employed.  **Thought records:** record their self-rated thought records starting from the fifth week of the intervention. The daily thought records include a series of questions related to psychological behaviors, potential solutions, the frequency and severity of these behaviors, etc.  **Pleasant events:** document pleasant events, they will be required to record both their personal pleasant experiences and those shared with others, and the content of the events, as well as the date and time. |

**Appendix C** Schedule and outcome assessments, according to SPIRIT 2013 and 2022 guidelines

| **Variable** | **Method and Timing** | **Measure (score range, direction, psychometrics, clinical targets)** |
| --- | --- | --- |
| **DESCRIPTIVES** | | |
| Sociodemographic | Self-report  T1 (week 0) | Age, gender, race/ethnicity, income, education, living arrangement, marital status, employment, financial pressure, hospitalization, social & leisure activities, religion, living habits, etc. Adapted from prior studies. |
| Medical history | Self-report  T1 (Week 0), T2 (Week 12) | Self-rated health, self-reported oral health and medical history: cardiovascular disease, cancer, stroke, kidney disease, arthritis, sleep apnea, neurological diseases, osteoporosis, vision, hearing, COVID-19, other chronic diseases, etc. |
| **PRIMARY OUTCOME** | | |
| Body weight | Interviewer measured  T1 (Week 0), T2 (Week 12) | Measured using the same electronic weight scale in the community center. Device is Segmental Body Composition Monitor BC-545N. |
| **CLINICAL** | | |
| Blood pressure | Interviewer measured  T1 (Week 0), T2 (Week 12) | Targets for the blood pressure component of MetS are: systolic ≥ 130 mmHg or diastolic ≥ 85 mmHg or on antihypertensive medication. Device is OMRON Premium Automatic Blood Pressure Monitor HEM-7211. |
| HDL Cholesterol | Fasting blood test  T1 (Week 0), T2 (Week 12) | Fasting blood sample analyzed by the Diagnostic & Laboratory Group. Targets for the HDL cholesterol < 40 mg/dL (1.03 mmol/L) in males and < 50 mg/dL (1.29 mmol/L) in females, or treatment for this lipid abnormality. |
| LDL Cholesterol |  | Targets for the LDL cholesterol ≥ 130 mg/dL (3.4 mmol/L) or treatment for this lipid abnormality. |
| Glucose |  | Targets for the fasting plasma glucose ≥ 100 mg/dL (5.6 mmol/L) or previously diagnosed type 2 diabetes. |
| Hemoglobin A1c |  | Targets for HbA1c ≥6.0%. Non-diabetics: 4-6%, Controlled diabetics: 6-8%. |
| Total blood cell count, creatinine, BUN, liver function test |  | Marker of diseases: anemia, diabetes, livers and kidney function. |
| Albumin & CRP |  | Nutritional marker and marker of inflammation. |

| **Variable** | **Method and Timing** | **Measure (score range, direction, psychometrics, clinical targets)** |
| --- | --- | --- |
| Homocysteine |  | Indicator of deficiency in vitamin B-12 or folate. |
| Pulse & heart rhythm | Interviewer-assessed  T1 (Week 0), T2 (Week 12) | OMRON Premium Automatic Blood Pressure Monitor HEM-7211. |
| Prescription  medications |  | Self-reported prescription medications, augmented by review of pill bottles or bags. |
| **ANTHROPOMETRIC CLINICAL PARAMETERS** | | |
| Waist circumference | Interviewer measured  T1 (Week 0), T2 (Week 12) | Measured with a clinic-used soft tape measure. Targets for the waist component of the MetS are: males ≥ 90 cm; females ≥ 80 cm. |
| Hand grip strength |  | Takei hand grip strength dynamometer GRIP-D, T.K.K.5401. |
| Calf circumference |  | Measured with a clinic-used soft tape measure. |
| Body fat  Total body water  Muscle mass  Physique rating  Bone mass  Basal metabolic rate  Metabolic age  Visceral fat level |  | Device is “Segmental Body Composition Monitor BC-545N”.  (<https://tanita.asia/?_page=products&_func=detail&_lang=en&_para%5B0%5D=11&_para%5B1%5D=44&_para%5B2%5D=243>)  -Body fat: the ideal body fat percentage ranges from 10% to 25% for males and from 20% to 32% for females. Typically, body fat percentage tends to increase with age.  -Total body water content is approximately 50% to 60% for males and 45% to 60% for females. This water content generally decreases with age, while a higher muscle mass is associated with greater water retention in the body.  -Muscle mass: higher levels of muscle mass are correlated with a reduced risk of developing diabetes in adulthood.  -Physique rating is categorized into nine body types, including: obese and overweight (1 = hidden obesity, 2 = obese, 3 = solid body), healthy (4 = active, 5 = normal, 6 = normal muscular), and fat-deficient (7 = slim, 8 = lean muscle, 9 = very muscular).  -Bone mass: for males, the average bone mass is approximately 2.5 kg for individuals weighing less than 60 kg, 2.5 kg for those weighing between 60 kg and 75 kg, and 3.2 kg for those weighing 75 kg or more. For females, the average bone mass is 1.8 kg for individuals weighing less than 45 kg, 2.2 kg for those weighing between 45 kg and 60 kg, and 2.5 kg for those weighing 60 kg or more.  -Basal metabolic rate (BMR) refers to the minimum daily energy expenditure, or caloric intake, required in a resting state (typically lying down) to support the functioning of essential organs, including the respiratory and circulatory systems, nervous system, liver, and kidneys. The daily basal metabolic energy requirement for adult males is approximately 1,200 to 1,600 calories, while for females, it ranges from 1,000 to 1,400 calories. |

| **Variable** | **Method and Timing** | **Measure (score range, direction, psychometrics, clinical targets)** |
| --- | --- | --- |
|  |  | -Metabolic age is defined as the age derived from comparing an individual's metabolic rate to the average BMR for their chronological age group. If an individual's metabolic age exceeds their actual age, it may indicate that current lifestyle and dietary practices should be reassessed. Increased PA can promote the development of healthy muscle tissue, thereby improving metabolic age.  -Visceral fat is closely associated with various health conditions, including heart disease, diabetes, and hypertension. Normal visceral fat levels are considered to be 4-6 for males and 2-4 for females. Values between 10 and 14 are classified as obese, while values ranging from 15 to 59 indicate a very high level of visceral fat, necessitating immediate consultation with a healthcare professional for intervention. |
| Body Mass Index (BMI) | Interviewer measured  T1 (Week 0), T2 (Week 12) | Device is Tanita Model - Segmental Body Composition Monitor BC-545N. Targets for BMI over 23. Obesity (defined as a BMI of ≥ 25.0 kg/m²) and overweight (BMI between 23.0 kg/m² and < 25.0 kg/m²). |
| **eHEALTH SYSTEM** | Home-based self-monitoring | Device is Model No.: OLN600. Variables include key health vitals: heart rate, respiratory rate, core body temperature, SpO2, and blood pressure, etc. |
| **SECONDARY OUTCOMES** | | |
| Intrinsic capacity | Interviewer measured  T1 (Week 0), T2 (Week 12) | -Cognitive state assessment includes memorizing three words (e.g., flower, rice, door); stating the current year, month, day, and location; and recalling the three words [2].  -Motor Function is assessed through two questions utilizing the chair stand test. Participants are asked to stand up from an armless chair five times without using their arms, and whether they can complete these five standing movements within 14 seconds, along with the total completion time [2].  -Vision: Do you have any problems with your eyes? Difficulties with farsightedness, reading, eye diseases, or are you currently receiving treatment (e.g. for diabetes or hypertension) [2] ?  -Hearing is assessed using the Whisper Test. The assessor stands an arm's length away from the participant while the participant presses the ear screen on the opposite ear to close it off. The assessor then whispers the numbers 2, 4, and 7 for the participant to repeat. If the participant hears clearly, they likely have normal hearing in that ear. Record these numbers, then move to the other side and test with different numbers (3, 6, and 8), recording these as well [2]. Psychological Well-Being is evaluated through two questions with binary 'yes' or 'no' responses [2]. Participants are asked if, in the past two weeks, they have experienced any of the following symptoms: (1) feeling down, depressed, or hopeless, and (2) having little interest or pleasure in doing things. |

| **Variable** | **Method and Timing** | **Measure (score range, direction, psychometrics, clinical targets)** |
| --- | --- | --- |
| Intrinsic capacity | Interviewer measured  T1 (Week 0), T2 (Week 12) | -Vitality (nutrition) is assessed using the 18-item Chinese version of the Mini-Nutritional Assessment (CMNA). This nutritional assessment questionnaire comprises 18 questions, with a maximum total score of 30 points [3]. A total score ranging from 24 to 30 points indicates normal nutritional status. Scores between 17 and 23.5 points suggest a risk of malnutrition, while a total score of less than 17 points indicates the presence of malnutrition.  -HRV serves as a reliable measure of the sympathetic and parasympathetic activity of the autonomic nervous system, providing insights into stress levels. Additionally, HRV is an important biomarker for assessing individuals’ psychological status, including anxiety. HRV is defined as the variation over time in the intervals (i.e., rhythm-to-rhythm intervals) between consecutive heartbeats, reflecting oscillations in instantaneous heart rates. When an individual is under stress, sympathetic activity typically increases while vagal activity decreases, resulting in an elevated heart rate and reduced HRV [4]. The normal range for HRV, with a confidence level, is typically reported as 0 to 100.  -HRR refers to the speed at which the heart rate decreases after exercise. It is a measure of CVD fitness and autonomic nervous system function, indicating how well the heart can return to its resting state following physical activity. HRR is a valuable metric for assessing CVD fitness and overall health and more sensitive at predicting the risk of MetS [5]. The normal range for HRR is reported as 40 - 220 bpm. |
| Sleep quality |  | The Chinese version of the Pittsburgh Sleep Quality Index (CPSQI) is designed to assess sleep quality and disturbances [6]. The first four questions pertain to sleeping habits over the past month: (1) What time do you usually go to bed at night? (2) After going to bed, how long do you typically lie awake before falling asleep? (3) What time do you usually wake up in the morning? and (4) Approximately how long do you spend actually asleep each night (which may differ from the time spent lying in bed)?  -In terms of sleep disturbances, the CPSQI includes nine questions with response options ranging from 0 (never) to 3 (three times a week or more). For instance, participants are asked, "In the past month, how many times has your sleep been disturbed by any of the following nine reasons?": difficulty falling asleep within 30 minutes, waking up during the night or early in the morning, needing to get up to use the bathroom, difficulty breathing, coughing or loud snoring, feeling excessively cold or hot, experiencing nightmares, and experiencing pain. A higher total score reflects greater sleep disruption. |

| **Variable** | **Method and Timing** | **Measure (score range, direction, psychometrics, clinical targets)** |
| --- | --- | --- |
| Sleep quality | Interviewer measured  T1 (Week 0), T2 (Week 12) | -Additionally, there are four questions related to sleep-related issues over the past month: (1) Did you need medication to help you sleep? (2) Did you find it difficult to stay awake while driving, eating, or engaging in daily social activities? (3) How much trouble did you have mustering the energy to complete your daily responsibilities? And (4) How would you rate your overall sleep quality in the past month? |
| Chronic pain |  | -The Wong-Baker Face Pain Rating Scale ranges from 0 to 10, with the following pain classifications: no pain (0-1), mild pain (1-3), moderate pain (4-6), and severe pain (7-10) [7].  -The 14-item Chinese version of the Brief Pain Inventory (CBPI) also uses a scale from 0 to 10, categorizing pain as mild (0-3), moderate (4-6), and severe (≥7) [8]. |
| Depressive symptoms |  | The 15-item Chinese version of the Geriatric Depression Scale (CGDS) utilizes a binary response format of 'yes' or 'no,' with each 'yes' response assigned a value of 1 point and each 'no' response assigned a value of 0 points [9]. The total score is calculated by summing the points for all items, with higher scores indicating a greater level of depression. The scoring categories are as follows: 0-5 indicates no depression; 6-9 indicates mild depression; and 10-15 indicates severe depression. |
| Perceived stress |  | The 14-item Chinese version of the Perceived Stress Scale (CPSS) yields scores ranging from 0 to 56, with response options from 0 (never) to 4 (often) [10]. Scores are calculated by reverse scoring the positively worded items (items 4, 5, 6, 7, 9, 10, and 13). The total score is obtained by summing the scores of all 14 items, with higher scores indicating greater perceived stress. |
| Self-care capacity |  | The 6-item Chinese version of Self-Care Scale (CSCS) employs a 3-point Likert scale, with responses ranging from 1 (independent) to 3 (dependent and unable to care for self) [2]. The total score can range from 6 to 18, with higher scores indicating a lower ability for self-care. |
| Social support |  | The 20-item Chinese version of the Medical Outcomes Study Social Support Survey (MOS-SSS-20) utilizes a 5-point Likert scale, with responses ranging from 1 (not at all) to 5 (all the time) [11]. The survey includes one subjective question to assess the size of the patient's social support network, along with 19 objective questions. These 19 items measure four dimensions of functional social support. Emotional/Informational Support: this dimension reflects the |

| **Variable** | **Method and Timing** | **Measure (score range, direction, psychometrics, clinical targets)** |
| --- | --- | --- |
| Social support | Interviewer measured  T1 (Week 0), T2 (Week 12) | expression of positive affect and the provision of advice, information, guidance, or feedback, comprising eight items (3, 4, 8, 9, 13, 16, 17, and 19). Instrumental Support: this dimension pertains to the provision of material or behavioral assistance, consisting of four items (2, 5, 12, and 15). Positive Social Interaction: this dimension captures the availability of others to engage in enjoyable activities, with four items (7, 11, 14, and 18). Affective Support: this dimension includes expressions of love and affection, represented by three items (6, 10, and 20). |
| **MEDIATORS** | | |
| Self-efficacy | Interviewer-assessed  T1 (Week 0), T2 (Week 12) | The 10-item Chinese version of the Generalized Self-Efficacy Scale (CGSE) employs a 4-point Likert response format, with options ranging from 0 (not at all true) to 4 (exactly true) [12]. The total score, which ranges from 10 to 40, is calculated by summing the scores of all 10 items. Higher total scores indicate greater levels of self-efficacy. |
| Digital health literacy |  | The 8-item Chinese version of the eHealth Literacy Scale (CeHEAL) utilizes a 5-point Likert scale, with response options ranging from 1 (strongly disagree) to 5 (strongly agree) [13, 14]. The total score, which ranges from 8 to 40, is calculated by summing the scores of all 8 items. Higher total scores reflect a greater perceived level of eHealth literacy. |
| Mindfulness |  | The 20-item Chinese version of the Five Facet Mindfulness Questionnaire (CFFMQ) employs a 5-point Likert scale, with response options ranging from 1 (never) to 5 (very often) [15]. Scores are derived by reverse scoring specific items (2, 4, 5, 6, 8, 13, 16, and 20). Each facet score is calculated as the average of four items, with higher scores (theoretical range = 1-5) indicating greater levels of positive thinking. |
|  |  | The 30-item Chinese version of the Nonattachment Scale (CNonattachment) employs a 6-point Likert scale, with responses ranging from 1 (strongly disagree) to 6 (strongly agree) [16]. The total score is computed by averaging the responses across all items, with higher scores reflecting a greater propensity for non-attachment. |

**References**

| **Variable** | **Method and Timing** | **Measure (score range, direction, psychometrics, clinical targets)** |
| --- | --- | --- |
| Gerotranscendence | Interviewer-assessed  T1 (Week 0), T2 (Week 12) | The 12-item Chinese version of the Self-Compassion Scale Short-Form (CSCS-SF) is calculated by summing the inverse scores for self-care items (2, 3, 5, 6, 7, and 10) and self-depreciation items (1, 4, 8, 9, 11, and 12) [17, 18]. Higher total scores indicate greater self-compassion, characterized by high levels of self-care and low levels of self-depreciation. |
|  |  | The 13-item Chinese version of the Sense of Coherence Scale (CSOC) utilizes a 7-point Likert response format [19]. The scale encompasses three domains: meaningfulness (items 1, 4, 7, and 12), manageability (items 3, 5, 10, and 13), and comprehensibility (items 2, 6, 8, 9, and 11). Scores are calculated by reverse scoring specific items (1, 2, 3, 7, and 10). Total scores range from 13 to 91, with higher scores indicating a stronger sense of coherence (SOC) and an enhanced ability to cope with stressful situations. |

1. Chi I, Boey KW. Hong Kong validation of measuring instruments of mental health status of the elderly. Clin Gerontol. 1993;13(4):35–51.
2. Leung AY, Su JJ, Lee ESH, Fung JT, Molassiotis A. Intrinsic capacity of older people in the community using WHO Integrated Care for Older People (ICOPE) framework: a cross-sectional study. BMC Geriatr. 2022;22(1):304.
3. Rubenstein LZ, Harker JO, Salvà A, Guigoz Y, Vellas B. Screening for undernutrition in geriatric practice: developing the short-form mini-nutritional assessment (MNA-SF). J Gerontol A Biol Sci Med Sci. 2001;56(6):M366–72.
4. Wong CL, Chien WT, Waye MMY, Szeto MWC, Li H. Nursing students' perceived anxiety and heart rate variability in mock skill competency assessment. PLoS One. 2023;18(10):e0293509.
5. Yu TY, Hong WJ, Jin SM, Hur KY, Jee JH, Bae JC, et al. Delayed heart rate recovery after exercise predicts development of metabolic syndrome: A retrospective cohort study. J Diabetes Investig. 2022;13(1):167-76.
6. Tsai PS, Wang SY, Wang MY, Su CT, Yang TT, Huang CJ, Fang SC. Psychometric evaluation of the Chinese version of the Pittsburgh Sleep Quality Index (CPSQI) in primary insomnia and control subjects. Qual Life Res. 2005;14(8):1943-52.
7. Kim EJ, Buschmann MT. Reliability and validity of the Faces Pain Scale with older adults. Int J Nurs Stud. 2006;43(4):447–56.
8. Ger LP, Ho ST, Sun WZ, Wang MS, Cleeland CS. Validation of the Brief Pain Inventory in a Taiwanese population. J Pain Symptom Manage. 1999;18(5):316–22.
9. Lai DW, Fung TS, Yuen CT. The factor structure of a Chinese version of the Geriatric Depression Scale. Int J Psychiatry Med. 2005;35(2):137–48.
10. Leung DY, Lam TH, Chan SS. Three versions of Perceived Stress Scale: validation in a sample of Chinese cardiac patients who smoke. BMC Public Health. 2010;10:513.
11. Yu DS, Lee DT, Woo J. Psychometric testing of the Chinese version of the medical outcomes study social support survey (MOS-SSS-C). Res Nurs Health. 2004;27(2):135-43.
12. Zeng G, Fung SF, Li JW, Hussain N, Yu P. Evaluating the psychometric properties and factor structure of the general self-efficacy scale in China. Curr Psychol. 2022;41:3970-80.
13. Norman CD, Skinner HA. eHEALS: the eHealth Literacy Scale. J Med Internet Res. 2006;8(4):e27.
14. Ma Z, Wu M. The psychometric properties of the Chinese eHealth Literacy Scale (C-eHEALS) in a Chinese rural population: cross-sectional validation study. J Med Internet Res. 2019;21(10):e15720.
15. Hou J, Wong SY, Lo HH, Mak WW, Ma HS. Validation of a Chinese version of the Five Facet Mindfulness Questionnaire in Hong Kong and development of a short form. Assess. 2014;21(3):363–71.
16. Sahdra BK, Shaver PR, Brown KW. A scale to measure nonattachment: a Buddhist complement to Western research on attachment and adaptive functioning. J Pers Assess. 2010;92(2):116-27.
17. Neff KD. Development and validation of a scale to measure self-compassion. Self Identity. 2003;2:223–50.
18. Raes F, Pommier E, Neff KD, Van Gucht D. Construction and factorial validation of a short form of the Self-Compassion Scale. Clin Psychol Psychother. 2011;18:250–5.
19. Wu BWY, Gao JL, Leung HK, Sik HH. A Randomized Controlled Trial of Awareness Training Program (ATP), a Group-Based Mahayana Buddhist Intervention. Mindfulness. 2019;10(7):1280-93.
